# Impact of asymptomatic *Cryptosporidium* infection on nutritional status in children under two: a multi-country cohort study

**DOI:** 10.1186/s40795-025-01183-2

**Published:** 2025-10-21

**Authors:** Murad Alam Khan, Md Ahshanul Haque, Md. Tariqujjaman, ASG Faruque, Tahmeed Ahmed, Mustafa Mahfuz

**Affiliations:** Nutrition Research Division, Mohakhali Dhaka, icddr,b 68,Shaheed Tajuddin Ahmed Sharani, 1212 Bangladesh

**Keywords:** Cryptosporidium, Malnutrition, Incidence rate, Poisson regression, MAL-ED study

## Abstract

**Background:**

Subclinical or asymptomatic cryptosporidiosis can contribute to malnutrition in children, especially in environments where the infection is persistent or occurs repeatedly. Despite the absence of symptoms, these infections are associated with impaired growth and nutritional deficiencies, potentially increasing the risk of stunting, wasting, and underweight. This study examined the association between asymptomatic *Cryptosporidium* infection and nutritional status in children under 2 years old.

**Methods:**

This is a secondary analysis of data from the MAL-ED study which is a longitudinal birth cohort study conducted in eight countries: Bangladesh, Brazil, India, Nepal, Pakistan, Peru, South Africa, and Tanzania, a multi-country investigation of enteric infections and malnutrition. We excluded Pakistan due to the lack of available anthropometric data. Nutritional indicators were assessed using the World Health Organization growth chart,[18] including stunting (length-for-age <-2 standard deviations [SD]), underweight (weight-for-age <-2 SD), and wasting (weight-for-length <-2 SD). Poisson regression estimated *Cryptosporidium incidence* rates, while GEE (generalized estimation equation) with a binomial family and logit link assessed associations with nutritional indicators, calculating odds ratios.

**Result:**

Findings indicate an incidence rate of 7.89 per 100 child-months for asymptomatic *Cryptosporidium* infections, which are significantly associated with stunting (aOR: 1.32; 95% CI: 1.24–1.41), wasting (aOR: 1.47; 95% CI: 1.08–1.49), and underweight (aOR: 1.47; 95% CI: 1.08–1.49). Incidence was highest in Tanzania (14.35) and Peru (12.55), indicating a hidden burden, while Brazil (1.34) had the lowest. Stunting was strongly associated with infection in India, Peru, and Tanzania, underweight in Bangladesh and Tanzania, and wasting was notably high in Peru.

**Conclusions:**

This study highlights the significant burden of asymptomatic *Cryptosporidium* infections and their potential impact on child growth and nutrition. The strong associations with stunting, wasting, and underweight suggest that even in the absence of symptoms, these infections may contribute to long-term developmental challenges. Furthermore, the differential effects on nutritional outcomes across countries emphasize the importance of targeted strategies to mitigate the hidden consequences of *Cryptosporidium* infections and improve child health globally.

## Introduction

Subclinical or asymptomatic cryptosporidiosis is a significant yet often overlooked contributor to childhood malnutrition, particularly in low-resource settings with poor sanitation and frequent exposure to enteric pathogens [1, 2]. Even without overt symptoms, *Cryptosporidium* can damage the intestinal lining, impair nutrient absorption, and hinder growth, leading to long-term developmental consequences such as stunting [3, 4].This impact is especially concerning in low- and middle-income countries (LMICs), where children face recurrent infections and co-exposure to multiple pathogens, compounding the risk of malnutrition [5].

Beyond its subclinical effects, *Cryptosporidium* is also a major cause of diarrhoeal disease, which remains a leading cause of childhood morbidity and mortality. In 2019, diarrhoeal disease accounted for about 9% of deaths among children under five, translating to over 1,300 deaths per day globally—many of which are preventable with basic treatment and sanitation [6, 7]. *Cryptosporidium* disproportionately affects young children, the immunocompromised, and those in impoverished environments, and has been identified as one of the top contributors to diarrhoea-related deaths [8]. Several studies have shown a significant association between cryptosporidiosis and undernutrition in children, though the mechanisms remain unclear and likely involve multiple interacting factors [9–12].In LMICs, where multi-pathogen infections are common, malnutrition often arises from a combination of enteric diseases rather than *Cryptosporidium* alone. Addressing this burden requires comprehensive strategies focused on both treatment and prevention, including improved sanitation, hygiene, and access to care.

The Etiology, Risk Factors, and Interactions of Enteric Infections and Malnutrition and the Consequences for Child Health (MAL-ED) Study was established at eight sites: Bangladesh, Brazil, India, Nepal, Pakistan, Peru, South Africa, and Tanzania, regions with historically high incidences of diarrhoeal disease and undernutrition [13]. Asymptomatic infection of *cryptosporidiosis*, a parasitic disease caused by *Cryptosporidium*, may significantly contribute to malnutrition and stunted growth over the long term [14]. This highlights the urgent need to address both malnutrition and diarrhoeal diseases in these regions. Moreover, previous studies have shown that *Cryptosporidium* infections can lead to growth faltering and cognitive deficits, particularly in children [15].

*Cryptosporidium* infections in young children even when asymptomatic pose a significant yet under recognized threat to child growth and nutrition, particularly in low- and middle-income countries (LMICs). While symptomatic cases are known to cause severe diarrhoea and malnutrition, subclinical infections often go undetected yet still contribute to growth faltering and stunting, perpetuating cycles of poverty and poor health. Landmark studies like the Global Enteric Multicenter Study (GEMS) [16] and the MAL-ED cohort have established that asymptomatic *Cryptosporidium* infections are linked to substantial deficits in linear growth during critical developmental windows. However, these studies primarily focused on symptomatic cases or limited geographic regions, leaving gaps in understanding the true burden of asymptomatic infections across diverse settings. Our study addresses this gap by analyzing multi-country data to determine location-specific incidence rates of asymptomatic *Cryptosporidium* and their association with nutritional outcomes. By doing so, we provide actionable evidence for policymakers to design targeted interventions that mitigate the hidden impact of this pathogen on child undernutrition.

## Method

The MAL-ED (Etiology, Risk Factors, and Interactions of Enteric Infections and Malnutrition) network is conducting a multi-country, longitudinal prospective cohort study to investigate the causes, risk factors, and interactions of enteric infections and malnutrition, along with their effects on child growth, cognitive development, and vaccine response. The MAL-ED study was conducted across 8 diverse sites: Bangladesh, Brazil, India, Nepal, Pakistan, Peru, South Africa, and Tanzania. The MAL-ED study focuses on birth cohorts that were followed longitudinally until 24 months of age across each of the 8 study sites. To support this, each site conducted a census of their local communities to assess the number of women of reproductive age and the population of children under five years old. At enrollment, the child’s date of birth, sex, and birth weight (if available) were recorded, along with breastfeeding initiation, length, weight, and head circumference. Twice-weekly home visits tracked infectious diseases, child health, and diet. Additional visits collected data on health, vaccinations, dietary intake, anthropometry, cognitive tests, and biological samples. Maternal and household characteristics were also documented. The MAL-ED study had inclusion and exclusion criteria.

The inclusion criteria: Healthy newborns enrolled within the first 17 days of life. Caregivers confirmed they did not intend to move out of the study area for at least six months after enrollment. Caregivers agreed to allow home visits twice a week.

The exclusion criteria: The family planned to leave the catchment area for more than 30 consecutive days during the first six months of follow-up. The mother was younger than 16 years of age. The mother already had another child enrolled in the MAL-ED cohort study. The child was part of a multiple birth (e.g., twins or triplets). The infant had evidence of serious illness, including: Hospitalization for reasons other than an uncomplicated healthy birth; A severe or chronic condition diagnosed by a physician (such as a neonatal disorder, or renal, liver, lung, heart, or congenital disease); or. Enteropathies diagnosed by a physician. The child’s guardian did not provide signed informed consent. The infant’s birth or enrollment weight was less than 1500 g.

**Fig. 1:**
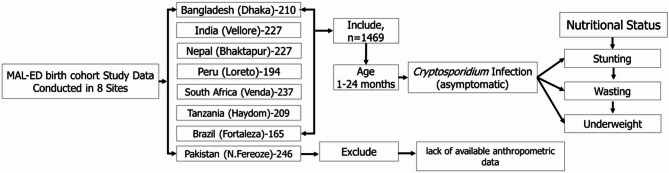
Selection of study participants

**Fig. 2:**
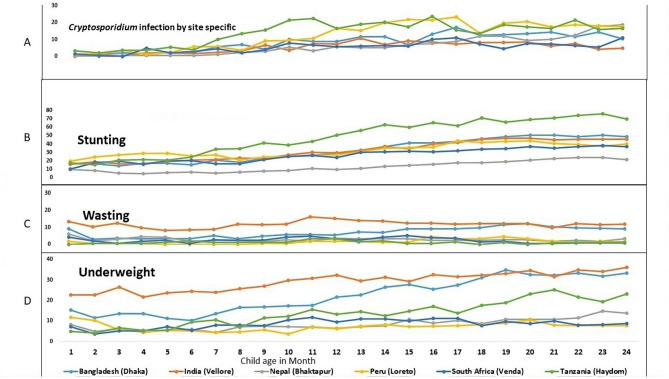
Site-specific prevalence of asymptomatic *Cryptosporidium* infection (%) of stunting, wasting and underweight

Further details of inclusion and exclusion criteria, sampling design, and data management have been described elsewhere [17]. For our analysis we have used secondary data from MAL-ED study network authority. We excluded Pakistan due to the lack of available anthropometric data. The design for this study has been selected for the children under 2 years of age, among them Bangladesh (210), Brazil (165), India (227), Nepal (227), Peru (194), South Africa (237), and Tanzania (209), our final analysis comprised 1469 participants (as shown in Fig. 1). The general characteristics of the final study population are presented in Table 1. A site-specific prevalence of stunting, wasting, underweight and asymptomatic *Cryptosporidium* infection have been shown in Fig. 2.

**Table 1:** General characteristics of MAL-ED study populations from November 2009 to February 2012

Characteristics, *n* (%)	Bangladesh *n* = 210	Brazil *n* = 165	India *n* = 227	Nepal *n* = 227	Peru *n* = 194	South Africa *n* = 237	Tanzania *n* = 209	Overall *n* = 1469
Male sex	108 (51.4)	89 (53.9)	105 (46.3)	122 (53.7)	105 (54.1)	120 (50.6)	105 (50.2)	754 (51.5)
Days of exclusive breastfeeding^†^	143.2 ± 42.7	93.7 ± 57.8	105.4 ± 42.9	92.5 ± 54.5	89.5 ± 61.3	38.6 ± 26.3	62.2 ± 35	89.3 ± 45.8
Birth weight (kg)^†^	2.8 ± 0.4	3.4 ± 0.5	2.9 ± 0.4	3 ± 0.4	3.1 ± 0.4	3.2 ± 0.5	3.2 ± 0.5	3.1 ± 0.4
Weight-for-age z-score at Enrollment^†^	−1.3 ± 0.9	−0.2 ± 1.0	−1.3 ± 1.0	−0.9 ± 1.0	−0.6 ± 0.9	−0.4 ± 1.0	−0.1 ± 0.9	−0.8 ± 1.1
Length-for-age z-score at Enrollment^†^	−0.96 ± 1.0	−0.8 ± 1.1	−1.0 ± 1.1	−0.7 ± 1	−0.9 ± 1	−0.7 ± 1.0.	−1 ± 1.1	−0.9 ± 1.1
Length-for-age z-score at 24 months^†^	−2.0 ± 0.9	0 ± 1.1	−1.9 ± 1	−1.3 ± 0.9	−1.9 ± 0.9	−1.7 ± 1.1	−2.7 ± 1	−1.6 ± 1.1
Maternal age (years)^†^	25.0 ± 5.0	25.4 ± 5.6	23.9 ± 4.2	26.6 ± 3.7	24.8 ± 6.3	27 ± 7.2	29.1 ± 6.5	26.1 ± 5.5
Maternal weight (kg)^†^	49.7 ± 8.5	62 ± 11.5	50.3 ± 9.3	56.2 ± 8.3	56.3 ± 9.6	68 ± 15.3	55.7 ± 8.8	56.9 ± 10.2
Maternal height (cm)^†^	149.0 ± 5.0	155.1 ± 6.7	151.1 ± 5.2	149.7 ± 5.3	150.2 ± 5.5	158.7 ± 6.6	155.9 ± 5.9	152.8 ± 5.7
Maternal educational level < 6 y	133 (63.3)	22 (13.3)	80 (35.2)	59 (26)	44 (22.7)	5 (2.1)	75 (35.9)	418 (28.4)
Mother has less than 3 living children	160 (76.2)	113 (68.5)	157 (69.8)	199 (87.7)	111 (57.2)	141 (59.5)	58 (27.8)	939 (5.74)
Ownership of chickens/ducks	3 (1.4)	1 (0.6)	14 (6.2)	73 (32.2)	75 (38.7)	87 (37.2)	204 (97.6)	457 (30.6)
Ownership of cows/bulls	1 (0.5)	-	5 (2.2)	3 (1.3)	-	33 (13.9)	157 (75.1)	199 (13.3)
Routine treatment of drinking water	130 (61.9)	10 (6.1)	7 (3.1)	98 (43.2)	32 (16.5)	12 (5.1)	12 (5.7)	301 (20.2)
Improved drinking water source	210 (100)	165 (100)	227 (100)	227 (100)	184 (94.9)	196 (82.7)	89 (42.6)	1298 (88.6)
Improved latrine	210 (100)	165 (100)	121 (53.3)	227 (100)	66 (34)	232 (97.9)	19 (9.1)	1040 (70.6)
Improved floor	204 (97.1)	165 (100)	222 (97.8)	109 (48)	69 (35.6)	231 (97.5)	13 (6.2)	1013 (68.9)
Monthly income <$150	69 (32.9)	161 (97.6)	19 (8.4)	106 (46.7)	58 (29.9)	179 (75.5)	0 (0)	592 (41.6)
WAMI score^†^	0.6 ± 0.1	0.8 ± 0.1	0.5 ± 0.2	0.7 ± 0.1	0.5 ± 0.1	0.8 ± 0.1	0.2 ± 0.1	0.6 ± 0.1

### Variable of interest

This study investigates the nutrition of children under 2 years of age. Nutritional indicators were assessed using anthropometric measures by following World Health Organization growth chart (18), including stunting (length-for-age z-score <−2), underweight (weight-for-age z-score <−2), and wasting (weight-for-length z-score <−2). These measures allowed for the classification of children into different categories based on their nutritional status. *Cryptosporidium* was the exposure variable in this study for assessing the relationship between asymptomatic *Cryptosporidium* and children’s nutritional status.

### Laboratory testing

Our community research team collected non-diarrhoeal stool samples on a monthly basis from same cohort children at multiple time points, without adding any fixative, and stored unprocessed stool aliquots in −80 °C freezers until further laboratory testing. The laboratory techniques were standardized and carefully synchronized across all participating laboratories at each study sites [19, 20]. To detect a total of 29 enteropathogens from a single sample, including *Cryptosporidium*,*shigella*,* enteroinvasive Escherichia coli (EIEC)*,* enteroaggregative Escherichia coli (EAEC)*,* Campylobacter jejuni/coli*,* and enterotoxigenic Escherichia coli (ETEC)*, we utilized the TaqMan Array Card *(*TAC*)* [21, 22], a customized and compartmentalized probe-based multiplex quantitative polymerase chain reaction system. The system employed standard optimized protocols for total nucleic acid extraction from stool samples, and the analytic limit was set at 35 cycles of threshold (Ct). A Ct value less than 35 was considered positive for all enteropathogens [23, 24]. The assays were done as part of the MAL-ED study; no further analysis was done.

### Statistical analyses

All analyses were conducted using STATA version 14 (Stata-Corp LP, College Station, TX) and Python 3.12 with PyCharm Community Edition 2023.3.4. Descriptive statistics included line graphs to illustrate site-specific prevalence of outcome variables, including *Cryptosporidium* infection. Frequency and percentage were employed to summarize qualitative variables, while mean and standard deviation were used for quantitative variables. To summarize *Cryptosporidium* infection at a single point after adjusting for time, Poisson regression was utilized to estimate site-specific incidence rates using the null model. Finally, generalized estimating equations (GEE) with a binomial family and logit link were employed to assess the association between nutritional indicators and *Cryptosporidium* infection, estimating odds ratios (OR) to determine the strength of the association. Adjusted odds ratios (aOR) were calculated using multiple GEE after controlling for relevant covariates, such as sex, WAMI score (water/sanitation, assets, maternal education, and income) [25], maternal height, the number of living children (fewer than three), and various pathogens including *Shigella*, *Enteroinvasive Escherichia coli* (EIEC), *Enteroaggregative Escherichia coli* (EAEC), *Campylobacter jejuni/coli*, and *Enterotoxigenic Escherichia coli* (ETEC), as well as the study site for overall estimates. Covariates were selected based on a literature review and our bivariate findings, with co-infections included in the model based on previous publications from the MAL-ED study.

## Result

Table 1 summarizes the general characteristics of the MAL-ED study population, which comprised 1,469 children aged 1–24 months across eight sites. The proportion of male participants was relatively consistent across countries, ranging from 46.3% in India to 54.1% in Peru. Maternal height varied notably, with South African mothers being the tallest on average (158.7 cm) and Bangladeshi mothers the shortest (149.0 cm), reflecting potential socio-economic and nutritional disparities. WAMI scores also differed, with Bangladesh, India, and Peru scoring lower (0.5–0.6) compared to Brazil and South Africa (0.8).

Figure 1 outlines the data extraction process and assesses the association between asymptomatic *Cryptosporidium* infection and nutritional status (stunting, wasting, and underweight), excluding participants with missing anthropometric data. In Fig. 2, (Panel A) highlights geographical and age-related variations in *Cryptosporidium* infections, revealing higher rates in children aged 12–18 months and certain regions. Panels B–D depict nutritional trends: stunting generally increases with age (6–24 months), wasting remains stable without significant trends, and underweight prevalence varies markedly by region, with India (Vellore) consistently showing higher rates.

**Fig. 3:**
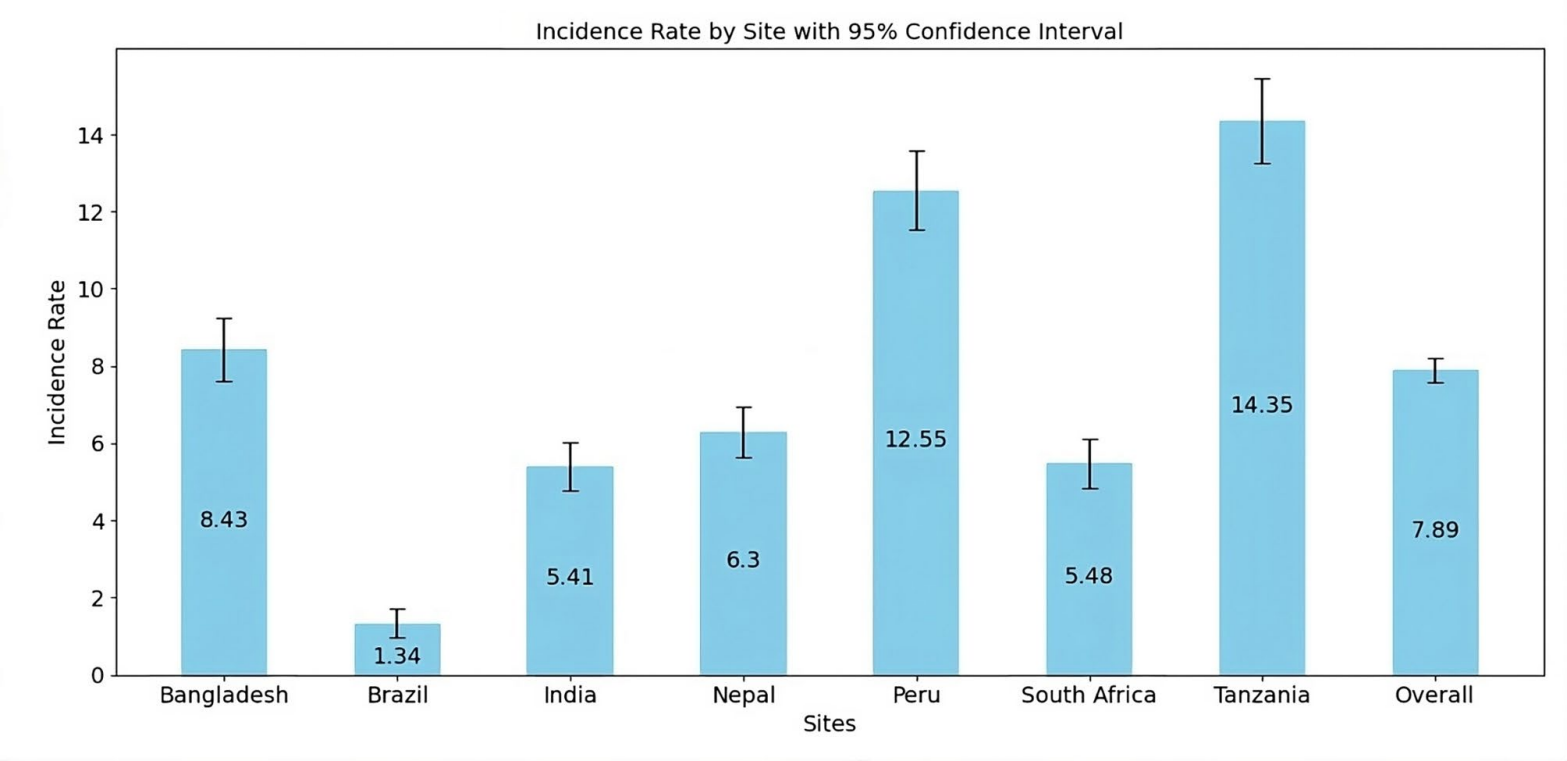
Site specific incidence rate of asymptomatic *Cryptosporidium* infection

Figure 3 illustrates that the overall incidence rate across all study sites was 7.89 per 100 child-months, with the highest rates observed in Tanzania (14.35) and Peru (12.55), highlighting a substantial burden of undetected infections that could still impact child health. In contrast, Brazil had the lowest incidence rate (1.34), suggesting a lower prevalence of these infections in that region.

Table 2 presents the adjusted associations between asymptomatic *Cryptosporidium* infection and different forms of malnutrition. Overall, infected children were more likely to be stunted (aOR: 1.29; 95% CI: 1.20–1.39; *p* < 0.001), wasted (aOR: 1.20; 95% CI: 1.01–1.43; *p* = 0.041), and underweight (aOR: 1.30; 95% CI: 1.19–1.42; *p* < 0.001).

**Table 2:** Associations between asymptomatic *Cryptosporidium* infection and children’s nutritional outcomes

	Crude OR (95% CI)	*P*-value	Adjusted OR (95% CI)	*P*-value
Stunting [LAZ<- 2]				
Overall	1.32 (1.24, 1.41)	0.000	1.29 (1.20, 1.39)	0.000
Bangladesh	1.27 (1.08, 1.49)	0.003	1.14 (0.95, 1.36)	0.152
Brazil	1.45 (0.45, 4.65)	0.535	1.64 (0.38, 7.08)	0.509
India	1.36 (1.14, 1.62)	0.001	1.37 (1.12, 1.66)	0.002
Nepal	1.33 (1.08, 1.65)	0.008	1.34 (1.05, 1.72)	0.180
Peru	1.24 (1.08, 1.42)	0.003	1.22 (1.05, 1.43)	0.001
South Africa	1.21 (0.99, 1.48)	0.063	1.19 (0.96, 1.48)	0.121
Tanzania	1.46 (1.28, 1.67)	0.000	1.38 (1.19, 1.60)	0.000
Wasting [WLZ<- 2]				
Overall	1.22 (1.02, 1.46)	0.028	1.2 (1.01, 1.43)	0.041
Bangladesh	1.18 (0.86, 1.62)	0.316	1.09 (0.78, 1.53)	0.600
Brazil	-	-	-	-
India	0.97 (0.72, 1.29)	0.821	0.95 (0.70, 1.28)	0.717
Nepal	1.23 (0.71, 2.14)	0.457	1.29 (0.72, 2.31)	0.391
Peru	1.89 (1.09, 3.25)	0.022	2.07 (1.15, 3.71)	0.015
South Africa	1.36 (0.72, 2.60)	0.345	1.34 (0.71, 2.55)	0.366
Tanzania	1.7 (0.88, 3.28)	0.113	1.66 (0.86, 3.23)	0.134
Underweight [WAZ<−2]				
Overall	1.32 (1.21, 1.43)	0.000	1.3 (1.19, 1.42)	0.000
Bangladesh	1.48 (1.26, 1.74)	0.000	1.37 (1.14, 1.64)	0.001
Brazil	-	-	-	-
India	1.08 (0.90, 1.29)	0.398	1.1 (0.91, 1.32)	0.332
Nepal	1.31 (1.02, 1.67)	0.031	1.3 (0.98, 1.74)	0.074
Peru	1.27 (1.01, 1.60)	0.041	1.27 (0.99, 1.64)	0.059
South Africa	1.30 (0.98, 1.74)	0.070	1.28 (0.94, 1.75)	0.123
Tanzania	1.42 (1.19, 1.69)	0.000	1.38 (1.14, 1.67)	0.001

Country-specific analysis showed a particularly strong link with stunting in India (aOR: 1.37; 95% CI: 1.12–1.66; *p* = 0.002) and Tanzania (aOR: 1.38; 95% CI: 1.19–1.60; *p* < 0.001). Peru also showed increased stunting (aOR: 1.22; 95% CI: 1.05–1.43; *p* = 0.001) along with a notably higher prevalence of wasting (aOR: 2.07; 95% CI: 1.15–3.71; *p* = 0.015). In Tanzania, underweight was significantly associated with infection (aOR: 1.38; 95% CI: 1.14–1.67; *p* < 0.001), as was also observed in Bangladesh (aOR: 1.37; 95% CI: 1.14–1.64; *p* < 0.001).

Overall, stunting appeared more strongly linked to infection in India, Peru, and Tanzania, while underweight was more pronounced in Bangladesh and Tanzania; Peru also showed a distinct concern with acute malnutrition reflected by higher wasting among infected children.

## Discussion

The main findings of this study are that asymptomatic *Cryptosporidium* infections are strongly associated with growth faltering and malnutrition in children under two years of age across diverse low- and middle-income settings, with an overall incidence rate of 7.89 infections per 100 child-months. Even without overt diarrhoea, infected children were more likely to be stunted, wasted, and underweight, showing how these silent infections can impair growth. Country-specific analyses highlight this burden: stunting was more strongly linked to infection in India (1.37), Peru (1.22), and Tanzania (1.38); Peru also had a notably higher prevalence of wasting (2.07), suggesting acute malnutrition is a particular concern there. Underweight was more pronounced in Bangladesh (1.37) and Tanzania (1.38), reflecting site-specific impacts. The highest infection rates were observed in Tanzania (14.35) and Peru (12.55), while Brazil (1.34) had the lowest. Rates peaked at 12–18 months, a critical window for growth faltering. Stunting generally increased from 6 to 24 months, wasting remained relatively stable, and underweight varied more across sites.

One of the paper from MAL-ED study has demonstrated *Cryptosporidium* infection with site-specific LAZ decline, notably in Bangladesh and India [26]. Moreover, some research has demonstrated a strong association between *Cryptosporidium* infection and growth faltering in children under 5 years old, with conditions like stunting, wasting, and underweight linked to the infection [27, 28]. Other studies focusing on children under the age of 5, have shown that pathogens such as *Shigella* [29], *Campylobacter* [30], and *Giardia* [31] also contribute significantly to malnutrition, alongside *Cryptosporidium* infections. Both treating the infection and improving nutritional status are essential, as malnutrition increases the risk of infection and worsens outcomes.

Malnutrition is a significant global health challenge, particularly in LMICs, where access to nutritious food is often limited. Inadequate nutrition can weaken the immune system, increasing susceptibility to infections [32]. While infections affect people regardless of their nutritional status, malnutrition can significantly worsen the severity of illness. In several districts of Bangladesh, stunting rates are high, reaching 38% in Sylhet [33], India has 35.5% stunting rate [34], 26.3% in Nepal [35], 13.1% in Peru [36], and 31.8 in Tanzania [37]. Addressing malnutrition and infectious diseases like *Cryptosporidium* requires a multi-faceted approach, including improving access to nutritious food and tackling underlying issues such as poverty, inadequate healthcare, and poor sanitation. Unsafe water and poor sanitation, identified as leading risk factors for childhood wasting and diarrhoea mortality, further highlight the need for comprehensive interventions to reduce both malnutrition and the prevalence of infection [38, 39].

Our findings on wasting are particularly striking, revealing that among all study sites, only the Peru site exhibits a compelling association between *Cryptosporidium* infection and the incidence of wasting. Inadequate nutrition profoundly impairs physical growth in undernourished children, particularly those residing in impoverished, low-income settings where chronic food insecurity and limited access to essential nutrients exacerbate developmental deficits and long-term health disparities [40, 41]. Evidence shows that in the first 1,000 days after birth, undernutrition hinders cognitive and physical development, leading to lifelong challenges and reduced potential [42]. *Cryptosporidium* infection, whether accompanied by diarrhoea or not, has a profound impact on nutrient absorption and intestinal digestion [43]. Some earlier studies suggest that *Cryptosporidium* infection impairs the growth and development [44]. These deficiencies weaken immunity and, over time, worsen nutritional status, creating a cycle of poor health and delayed recovery [45]. Our findings demonstrate a significant correlation with underweight, identifying Bangladesh, Nepal, Peru, and Tanzania as the countries disproportionately affected, underscoring their status as critical hotspots for this nutritional challenge. Understanding these regional differences is important for developing targeted interventions to address malnutrition and reduce the risk of infection.

The key strength of this study is its focus on children up to 2 years of age, a critical period for growth and development, whereas most other studies on *Cryptosporidium* infection and growth faltering have primarily targeted children under 5 years old [46]. The findings of this study are pivotal, highlighting the critical importance of the first two years of life as a vital period for growth and development, during which children are especially susceptible to infections and malnutrition, thereby increasing the risk of enduring consequences such as stunting, wasting, and underweight. The prospective longitudinal cohort study on the causes, risk factors, and interactions of enteric infections and malnutrition, such as the MAL-ED study, reinforces this by demonstrating how chronic exposure to enteric pathogens like *Cryptosporidium* can contribute to growth faltering.

Although the study has notable strengths, it also presents several limitations, such as its focus on asymptomatic *Cryptosporidium* infections, which might underestimate the burden of symptomatic cases. The association between asymptomatic *Cryptosporidium* infections and growth measurements suggests potential growth impacts even without clinical symptoms. However, other factors, including the presence of multiple pathogens in the same host, may also contribute to these outcomes. Co-infections with other pathogens and key environmental factors, such as access to clean water, were not fully accounted for, potentially confounding the associations with malnutrition. Additionally, the reliance on anthropometric measures does not capture the complexity of nutritional status, including micronutrient deficiencies. Variability in health systems may have affected infection detection, and findings may not be generalizable to all LMICs.

These findings highlight the need for early screening of *Cryptosporidium* in high-risk settings, even in the absence of symptoms, to support timely nutritional interventions. Strengthening WASH (water, sanitation, and hygiene) programs is essential to prevent transmission, especially in regions with high malnutrition burdens. Integrated strategies combining infection control and nutrition support are critical to improving child health outcomes in LMICs.

The study highlights the vulnerability of children in their first two years of life to infections and malnutrition, emphasizing the need for early interventions to prevent issues like stunting, wasting, and underweight. Additionally, future research should address the study’s limitations and investigate the mechanisms underlying the relationship between asymptomatic *Cryptosporidium* and child growth and development. Understanding these mechanisms will be essential for developing effective prevention and treatment strategies for asymptomatic *Cryptosporidium*, particularly in populations with high rates of child malnutrition.

## Conclusion

The study found a significant association between asymptomatic *Cryptosporidium* infection and malnutrition, particularly stunting, wasting, and underweight among children under two years age. The strong associations with stunting, wasting, and underweight suggest that even in the absence of symptoms, these infections may contribute to long-term developmental challenges. The geographic variation in incidence rates, with Tanzania and Peru experiencing the highest burden and Brazil the lowest, underscores the need for region-specific public health interventions. Furthermore, the differential effects on nutritional outcomes across countries emphasize the importance of targeted strategies to mitigate the hidden consequences of *Cryptosporidium* infections and improve child health globally.

## Data Availability

All relevant data, including personal data, is available upon request from the ClinEpiDB database ([https://clinepidb.org/ce/app/record/dataset/DS\_3dbf92dc05](https:/clinepidb.org/ce/app/record/dataset/DS_3dbf92dc05)). We received data access over a formal requestfrom the ClinEpiDB. For data assistance, contact Dr. Mustafa Mahfuz (mustafa@icddrb.org)”.

## Declarations

Written informed consent was obtained from the parents or legal guardians of each child prior to data collection. All methods were performed in accordance with relevant guidelines and regulations, and the study was conducted in compliance with the principles of the Declaration of Helsinki(WMA, 2013). All assays were conducted as part of the MAL-ED study, with no additional assays or sample collection performed.

### Author information

Murad Alam Khan, Md. Ahshanul Haque contributed equally to this work.Md. Tariqujjaman, ASG Faruque, Tahmeed Ahmed, Mustafa Mahfuz, agreed to review the final version of the manuscript.
